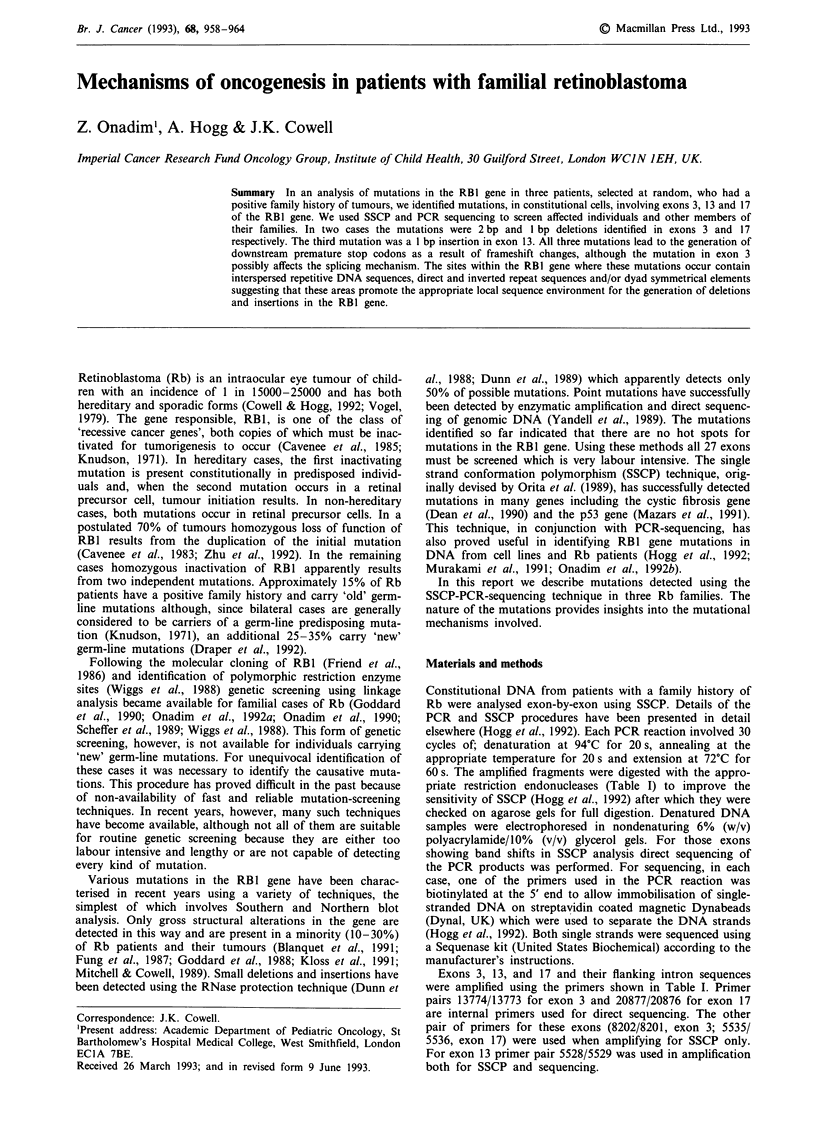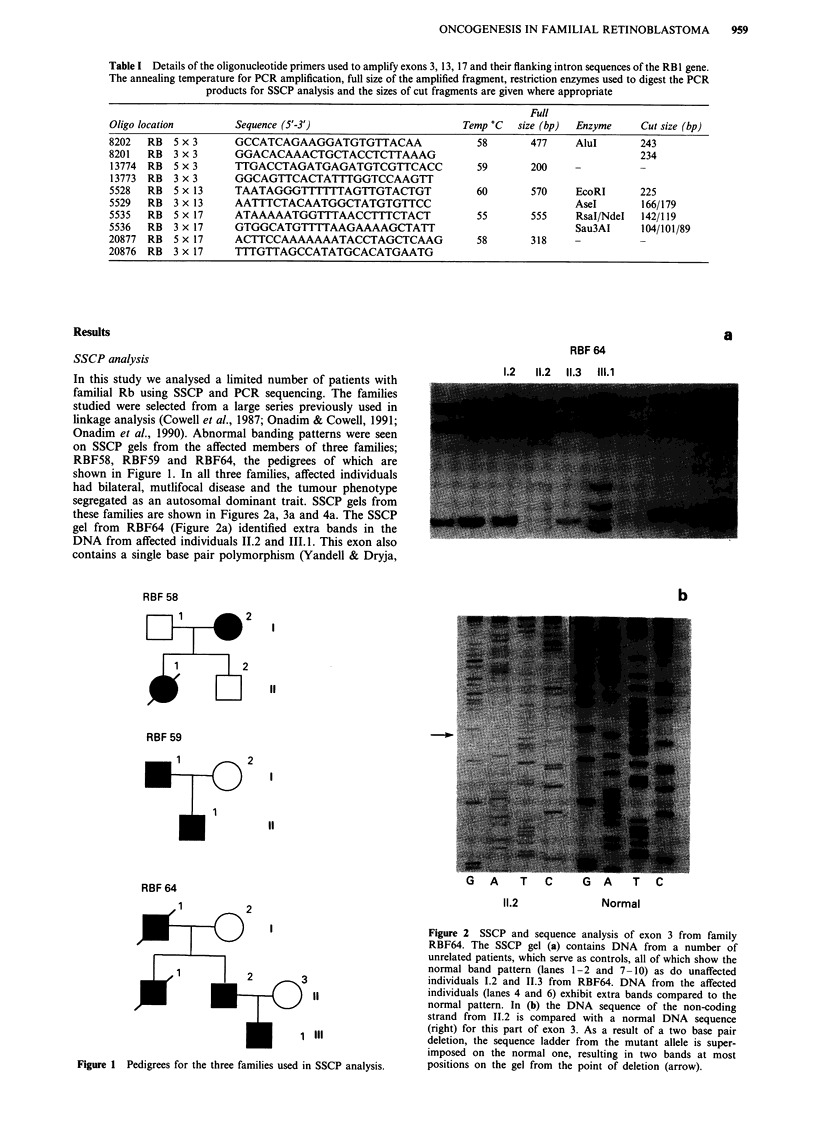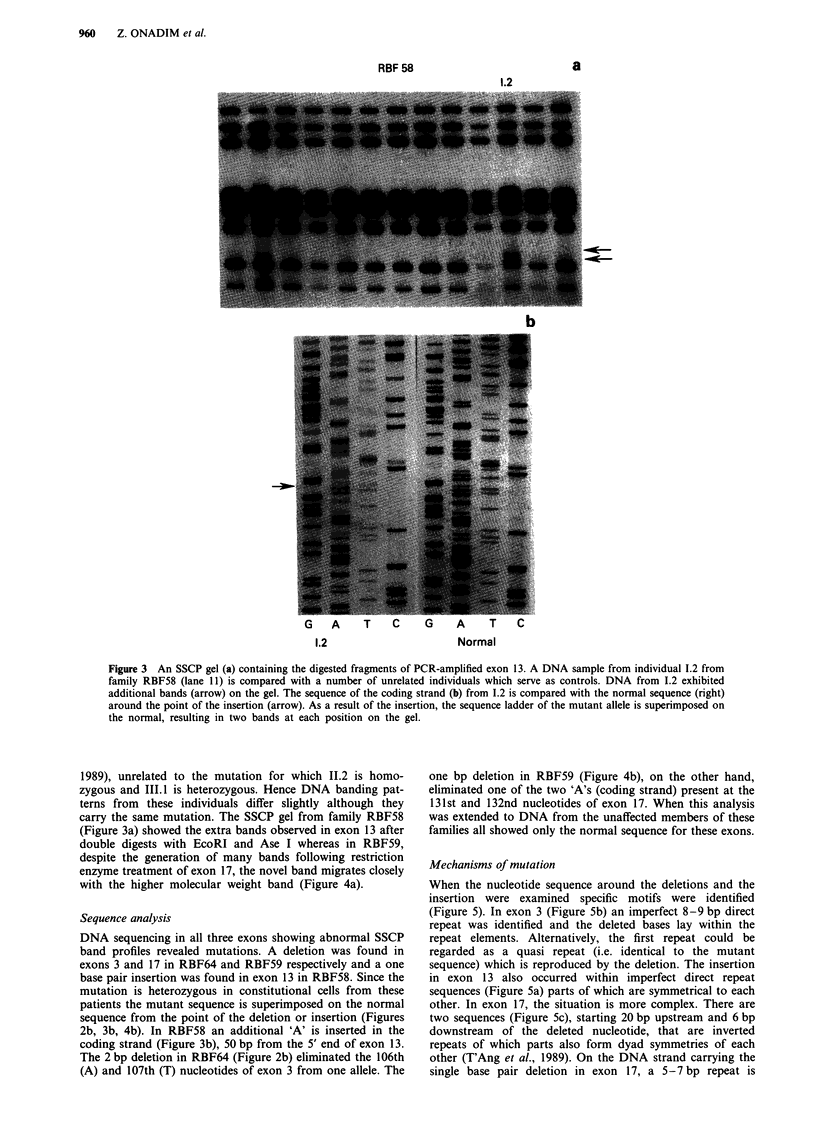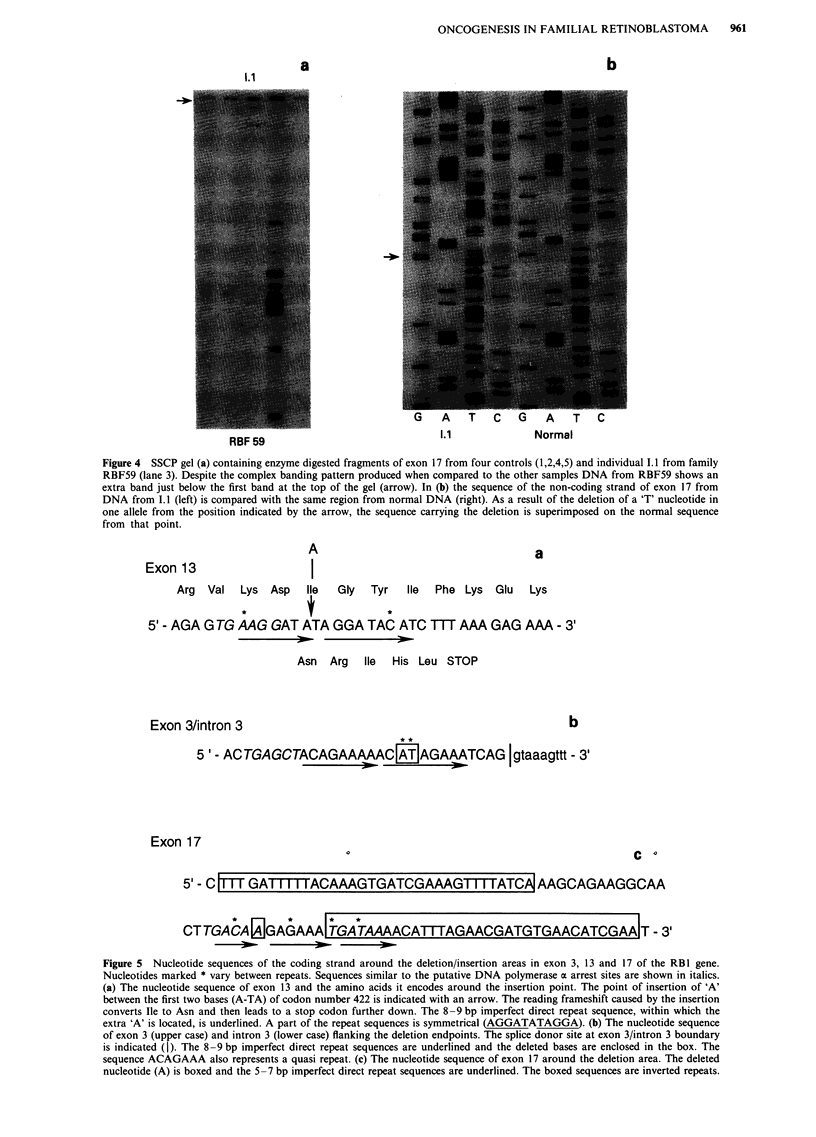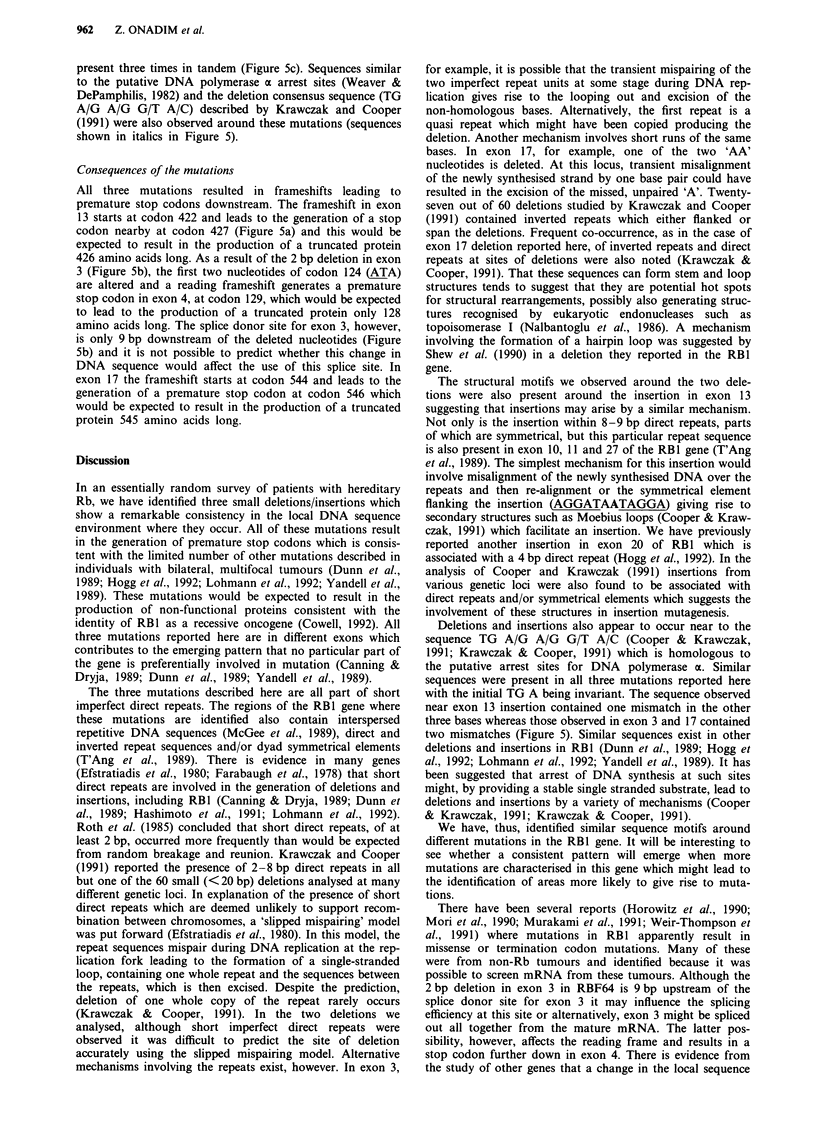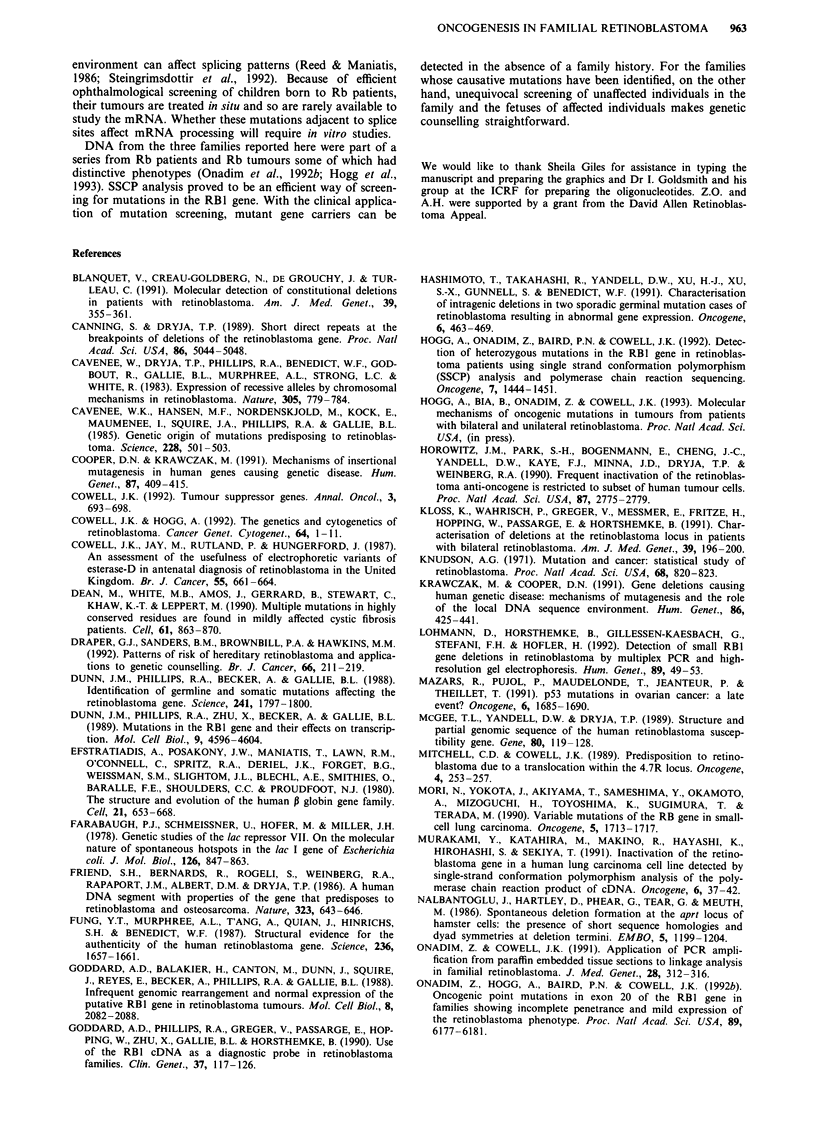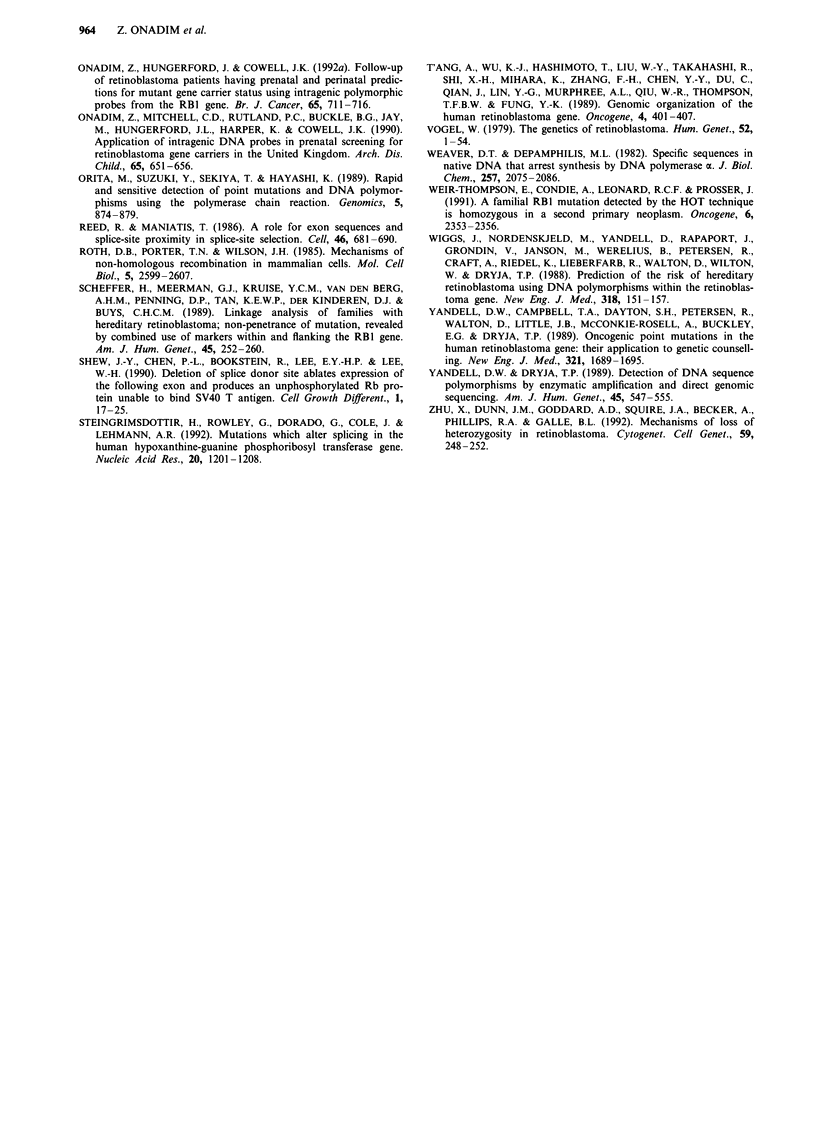# Mechanisms of oncogenesis in patients with familial retinoblastoma.

**DOI:** 10.1038/bjc.1993.461

**Published:** 1993-11

**Authors:** Z. Onadim, A. Hogg, J. K. Cowell

**Affiliations:** Imperial Cancer Research Fund Oncology Group, Institute of Child Health, London, UK.

## Abstract

**Images:**


*Br. J. Cancer* (1993), **68**, 958–964 © Macmillan Press Ltd., 1993

### Mechanisms of oncogenesis in patients with familial retinoblastoma

Z. Onadim[1], A. Hogg & J.K. Cowell

*Imperial Cancer Research Fund Oncology Group, Institute of Child Health, 30 Guilford Street, London WC1N 1EH, UK.*

**Summary** In an analysis of mutations in the RB1 gene in three patients, selected at random, who had a positive family history of tumours, we identified mutations, in constitutional cells, involving exons 3, 13 and 17 of the RB1 gene. We used SSCP and PCR sequencing to screen affected individuals and other members of their families. In two cases the mutations were 2 bp and 1 bp deletions identified in exons 3 and 17 respectively. The third mutation was a 1 bp insertion in exon 13. All three mutations lead to the generation of downstream premature stop codons as a result of frameshift changes, although the mutation in exon 3 possibly affects the splicing mechanism. The sites within the RB1 gene where these mutations occur contain interspersed repetitive DNA sequences, direct and inverted repeat sequences and/or dyad symmetrical elements suggesting that these areas promote the appropriate local sequence environment for the generation of deletions and insertions in the RB1 gene.

Retinoblastoma (Rb) is an intraocular eye tumour of children with an incidence of 1 in 15000–25000 and has both hereditary and sporadic forms (Cowell & Hogg, 1992; Vogel, 1979). The gene responsible, RB1, is one of the class of 'recessive cancer genes', both copies of which must be inactivated for tumorigenesis to occur (Cavenee *et al.*, 1985; Knudson, 1971). In hereditary cases, the first inactivating mutation is present constitutionally in predisposed individuals and, when the second mutation occurs in a retinal precursor cell, tumour initiation results. In non-hereditary cases, both mutations occur in retinal precursor cells. In a postulated 70% of tumours homozygous loss of function of RB1 results from the duplication of the initial mutation (Cavenee *et al.*, 1983; Zhu *et al.*, 1992). In the remaining cases homozygous inactivation of RB1 apparently results from two independent mutations. Approximately 15% of Rb patients have a positive family history and carry 'old' germ-line mutations although, since bilateral cases are generally considered to be carriers of a germ-line predisposing mutation (Knudson, 1971), an additional 25–35% carry 'new' germ-line mutations (Draper *et al.*, 1992).

Following the molecular cloning of RB1 (Friend *et al.*, 1986) and identification of polymorphic restriction enzyme sites (Wiggs *et al.*, 1988) genetic screening using linkage analysis became available for familial cases of Rb (Goddard *et al.*, 1990; Onadim *et al.*, 1992*a*; Onadim *et al.*, 1990; Scheffer *et al.*, 1989; Wiggs *et al.*, 1988). This form of genetic screening, however, is not available for individuals carrying 'new' germ-line mutations. For unequivocal identification of these cases it was necessary to identify the causative mutations. This procedure has proved difficult in the past because of non-availability of fast and reliable mutation-screening techniques. In recent years, however, many such techniques have become available, although not all of them are suitable for routine genetic screening because they are either too labour intensive and lengthy or are not capable of detecting every kind of mutation.

Various mutations in the RB1 gene have been characterised in recent years using a variety of techniques, the simplest of which involves Southern and Northern blot analysis. Only gross structural alterations in the gene are detected in this way and are present in a minority (10–30%) of Rb patients and their tumours (Blanquet *et al.*, 1991; Fung *et al.*, 1987; Goddard *et al.*, 1988; Kloss *et al.*, 1991; Mitchell & Cowell, 1989). Small deletions and insertions have been detected using the RNase protection technique (Dunn *et al.*, 1988; Dunn *et al.*, 1989) which apparently detects only 50% of possible mutations. Point mutations have successfully been detected by enzymatic amplification and direct sequencing of genomic DNA (Yandell *et al.*, 1989). The mutations identified so far indicated that there are no hot spots for mutations in the RB1 gene. Using these methods all 27 exons must be screened which is very labour intensive. The single strand conformation polymorphism (SSCP) technique, originally devised by Orita *et al.* (1989), has successfully detected mutations in many genes including the cystic fibrosis gene (Dean *et al.*, 1990) and the p53 gene (Mazars *et al.*, 1991). This technique, in conjunction with PCR-sequencing, has also proved useful in identifying RB1 gene mutations in DNA from cell lines and Rb patients (Hogg *et al.*, 1992; Murakami *et al.*, 1991; Onadim *et al.*, 1992*b*).

In this report we describe mutations detected using the SSCP-PCR-sequencing technique in three Rb families. The nature of the mutations provides insights into the mutational mechanisms involved.

### Materials and methods

Constitutional DNA from patients with a family history of Rb were analysed exon-by-exon using SSCP. Details of the PCR and SSCP procedures have been presented in detail elsewhere (Hogg *et al.*, 1992). Each PCR reaction involved 30 cycles of; denaturation at 94°C for 20 s, annealing at the appropriate temperature for 20 s and extension at 72°C for 60 s. The amplified fragments were digested with the appropriate restriction endonucleases (Table I) to improve the sensitivity of SSCP (Hogg *et al.*, 1992) after which they were checked on agarose gels for full digestion. Denatured DNA samples were electrophoresed in nondenaturing 6% (w/v) polyacrylamide/10% (v/v) glycerol gels. For those exons showing band shifts in SSCP analysis direct sequencing of the PCR products was performed. For sequencing, in each case, one of the primers used in the PCR reaction was biotinylated at the 5′ end to allow immobilisation of single-stranded DNA on streptavidin coated magnetic Dynabeads (Dynal, UK) which were used to separate the DNA strands (Hogg *et al.*, 1992). Both single strands were sequenced using a Sequenase kit (United States Biochemical) according to the manufacturer's instructions.

Exons 3, 13, and 17 and their flanking intron sequences were amplified using the primers shown in Table I. Primer pairs 13774/13773 for exon 3 and 20877/20876 for exon 17 are internal primers used for direct sequencing. The other pair of primers for these exons (8202/8201, exon 3; 5535/5536, exon 17) were used when amplifying for SSCP only. For exon 13 primer pair 5528/5529 was used in amplification both for SSCP and sequencing.

Correspondence: J.K. Cowell.
[1]Present address: Academic Department of Pediatric Oncology, St Bartholomew's Hospital Medical College, West Smithfield, London EC1A 7BE.
Received 26 March 1993; and in revised form 9 June 1993.

**Table I** Details of the oligonucleotide primers used to amplify exons 3, 13, 17 and their flanking intron sequences of the RB1 gene. The annealing temperature for PCR amplification, full size of the amplified fragment, restriction enzymes used to digest the PCR products for SSCP analysis and the sizes of cut fragments are given where appropriate

| *Oligo location* | | | *Sequence (5′-3′)* | *Temp °C* | *Full size (bp)* | *Enzyme* | *Cut size (bp)* |
|---|---|---|---|---|---|---|---|
| 8202 | RB | 5 × 3 | GCCATCAGAAGGATGTGTTACAA | 58 | 477 | AluI | 243 |
| 8201 | RB | 3 × 3 | GGACACAAACTGCTACCTCTTAAAG | | | | 234 |
| 13774 | RB | 5 × 3 | TTGACCTAGATGAGATGTCGTTCACC | 59 | 200 | – | – |
| 13773 | RB | 3 × 3 | GGCAGTTCACTATTTGGTCCAAGTT | | | | |
| 5528 | RB | 5 × 13 | TAATAGGGTTTTTTAGTTGTACTGT | 60 | 570 | EcoRI | 225 |
| 5529 | RB | 3 × 13 | AATTTCTACAATGGCTATGTGTTCC | | | AseI | 166/179 |
| 5535 | RB | 5 × 17 | ATAAAAATGGTTTAACCTTTCTACT | 55 | 555 | RsaI/NdeI | 142/119 |
| 5536 | RB | 3 × 17 | GTGGCATGTTTTAAGAAAAGCTATT | | | Sau3AI | 104/101/89 |
| 20877 | RB | 5 × 17 | ACTTCCAAAAAAAATACCTAGCTCAAG | 58 | 318 | – | – |
| 20876 | RB | 3 × 17 | TTTGTTAGCCATATGCACATGAATG | | | | |

### Results

### *SSCP analysis*

In this study we analysed a limited number of patients with familial Rb using SSCP and PCR sequencing. The families studied were selected from a large series previously used in linkage analysis (Cowell *et al.*, 1987; Onadim & Cowell, 1991; Onadim *et al.*, 1990). Abnormal banding patterns were seen on SSCP gels from the affected members of three families; RBF58, RBF59 and RBF64, the pedigrees of which are shown in Figure 1. In all three families, affected individuals had bilateral, mutlifocal disease and the tumour phenotype segregated as an autosomal dominant trait. SSCP gels from these families are shown in Figures 2a, 3a and 4a. The SSCP gel from RBF64 (Figure 2a) identified extra bands in the DNA from affected individuals II.2 and III.1. This exon also contains a single base pair polymorphism (Yandell & Dryja,

**Figure 1** Pedigrees for the three families used in SSCP analysis.

**Figure 2** SSCP and sequence analysis of exon 3 from family RBF64. The SSCP gel (**a**) contains DNA from a number of unrelated patients, which serve as controls, all of which show the normal band pattern (lanes 1–2 and 7–10) as do unaffected individuals I.2 and II.3 from RBF64. DNA from the affected individuals (lanes 4 and 6) exhibit extra bands compared to the normal pattern. In (**b**) the DNA sequence of the non-coding strand from II.2 is compared with a normal DNA sequence (right) for this part of exon 3. As a result of a two base pair deletion, the sequence ladder from the mutant allele is superimposed on the normal one, resulting in two bands at most positions on the gel from the point of deletion (arrow).

**Figure 3** An SSCP gel (**a**) containing the digested fragments of PCR-amplified exon 13. A DNA sample from individual I.2 from family RBF58 (lane 11) is compared with a number of unrelated individuals which serve as controls. DNA from I.2 exhibited additional bands (arrow) on the gel. The sequence of the coding strand (**b**) from I.2 is compared with the normal sequence (right) around the point of the insertion (arrow). As a result of the insertion, the sequence ladder of the mutant allele is superimposed on the normal, resulting in two bands at each position on the gel.

1989), unrelated to the mutation for which II.2 is homozygous and III.1 is heterozygous. Hence DNA banding patterns from these individuals differ slightly although they carry the same mutation. The SSCP gel from family RBF58 (Figure 3a) showed the extra bands observed in exon 13 after double digests with EcoRI and Ase I whereas in RBF59, despite the generation of many bands following restriction enzyme treatment of exon 17, the novel band migrates closely with the higher molecular weight band (Figure 4a).

*Sequence analysis*

DNA sequencing in all three exons showing abnormal SSCP band profiles revealed mutations. A deletion was found in exons 3 and 17 in RBF64 and RBF59 respectively and a one base pair insertion was found in exon 13 in RBF58. Since the mutation is heterozygous in constitutional cells from these patients the mutant sequence is superimposed on the normal sequence from the point of the deletion or insertion (Figures 2b, 3b, 4b). In RBF58 an additional 'A' is inserted in the coding strand (Figure 3b), 50 bp from the 5′ end of exon 13. The 2 bp deletion in RBF64 (Figure 2b) eliminated the 106th (A) and 107th (T) nucleotides of exon 3 from one allele. The one bp deletion in RBF59 (Figure 4b), on the other hand, eliminated one of the two 'A's (coding strand) present at the 131st and 132nd nucleotides of exon 17. When this analysis was extended to DNA from the unaffected members of these families all showed only the normal sequence for these exons.

*Mechanisms of mutation*

When the nucleotide sequence around the deletions and the insertion were examined specific motifs were identified (Figure 5). In exon 3 (Figure 5b) an imperfect 8–9 bp direct repeat was identified and the deleted bases lay within the repeat elements. Alternatively, the first repeat could be regarded as a quasi repeat (i.e. identical to the mutant sequence) which is reproduced by the deletion. The insertion in exon 13 also occurred within imperfect direct repeat sequences (Figure 5a) parts of which are symmetrical to each other. In exon 17, the situation is more complex. There are two sequences (Figure 5c), starting 20 bp upstream and 6 bp downstream of the deleted nucleotide, that are inverted repeats of which parts also form dyad symmetries of each other (T'Ang *et al.*, 1989). On the DNA strand carrying the single base pair deletion in exon 17, a 5–7 bp repeat is

**Figure 4** SSCP gel (**a**) containing enzyme digested fragments of exon 17 from four controls (1,2,4,5) and individual I.1 from family RBF59 (lane 3). Despite the complex banding pattern produced when compared to the other samples DNA from RBF59 shows an extra band just below the first band at the top of the gel (arrow). In (**b**) the sequence of the non-coding strand of exon 17 from DNA from I.1 (left) is compared with the same region from normal DNA (right). As a result of the deletion of a 'T' nucleotide in one allele from the position indicated by the arrow, the sequence carrying the deletion is superimposed on the normal sequence from that point.

**Figure 5** Nucleotide sequences of the coding strand around the deletion/insertion areas in exon 3, 13 and 17 of the RB1 gene. Nucleotides marked * vary between repeats. Sequences similar to the putative DNA polymerase α arrest sites are shown in italics. (**a**) The nucleotide sequence of exon 13 and the amino acids it encodes around the insertion point. The point of insertion of 'A' between the first two bases (A-TA) of codon number 422 is indicated with an arrow. The reading frameshift caused by the insertion converts Ile to Asn and then leads to a stop codon further down. The 8–9 bp imperfect direct repeat sequence, within which the extra 'A' is located, is underlined. A part of the repeat sequences is symmetrical (AGGATATAGGA). (**b**) The nucleotide sequence of exon 3 (upper case) and intron 3 (lower case) flanking the deletion endpoints. The splice donor site at exon 3/intron 3 boundary is indicated (|). The 8–9 bp imperfect direct repeat sequences are underlined and the deleted bases are enclosed in the box. The sequence ACAGAAA also represents a quasi repeat. (**c**) The nucleotide sequence of exon 17 around the deletion area. The deleted nucleotide (A) is boxed and the 5–7 bp imperfect direct repeat sequences are underlined. The boxed sequences are inverted repeats.

present three times in tandem (Figure 5c). Sequences similar to the putative DNA polymerase α arrest sites (Weaver & DePamphilis, 1982) and the deletion consensus sequence (TG A/G A/G G/T A/C) described by Krawczak and Cooper (1991) were also observed around these mutations (sequences shown in italics in Figure 5).

*Consequences of the mutations*

All three mutations resulted in frameshifts leading to premature stop codons downstream. The frameshift in exon 13 starts at codon 422 and leads to the generation of a stop codon nearby at codon 427 (Figure 5a) and this would be expected to result in the production of a truncated protein 426 amino acids long. As a result of the 2 bp deletion in exon 3 (Figure 5b), the first two nucleotides of codon 124 (ATA) are altered and a reading frameshift generates a premature stop codon in exon 4, at codon 129, which would be expected to lead to the production of a truncated protein only 128 amino acids long. The splice donor site for exon 3, however, is only 9 bp downstream of the deleted nucleotides (Figure 5b) and it is not possible to predict whether this change in DNA sequence would affect the use of this splice site. In exon 17 the frameshift starts at codon 544 and leads to the generation of a premature stop codon at codon 546 which would be expected to result in the production of a truncated protein 545 amino acids long.

**Discussion**

In an essentially random survey of patients with hereditary Rb, we have identified three small deletions/insertions which show a remarkable consistency in the local DNA sequence environment where they occur. All of these mutations result in the generation of premature stop codons which is consistent with the limited number of other mutations described in individuals with bilateral, multifocal tumours (Dunn *et al.*, 1989; Hogg *et al.*, 1992; Lohmann *et al.*, 1992; Yandell *et al.*, 1989). These mutations would be expected to result in the production of non-functional proteins consistent with the identity of RB1 as a recessive oncogene (Cowell, 1992). All three mutations reported here are in different exons which contributes to the emerging pattern that no particular part of the gene is preferentially involved in mutation (Canning & Dryja, 1989; Dunn *et al.*, 1989; Yandell *et al.*, 1989).

The three mutations described here are all part of short imperfect direct repeats. The regions of the RB1 gene where these mutations are identified also contain interspersed repetitive DNA sequences (McGee *et al.*, 1989), direct and inverted repeat sequences and/or dyad symmetrical elements (T'Ang *et al.*, 1989). There is evidence in many genes (Efstratiadis *et al.*, 1980; Farabaugh *et al.*, 1978) that short direct repeats are involved in the generation of deletions and insertions, including RB1 (Canning & Dryja, 1989; Dunn *et al.*, 1989; Hashimoto *et al.*, 1991; Lohmann *et al.*, 1992). Roth *et al.* (1985) concluded that short direct repeats, of at least 2 bp, occurred more frequently than would be expected from random breakage and reunion. Krawczak and Cooper (1991) reported the presence of 2–8 bp direct repeats in all but one of the 60 small (<20 bp) deletions analysed at many different genetic loci. In explanation of the presence of short direct repeats which are deemed unlikely to support recombination between chromosomes, a 'slipped mispairing' model was put forward (Efstratiadis *et al.*, 1980). In this model, the repeat sequences mispair during DNA replication at the replication fork leading to the formation of a single-stranded loop, containing one whole repeat and the sequences between the repeats, which is then excised. Despite the prediction, deletion of one whole copy of the repeat rarely occurs (Krawczak & Cooper, 1991). In the two deletions we analysed, although short imperfect direct repeats were observed it was difficult to predict the site of deletion accurately using the slipped mispairing model. Alternative mechanisms involving the repeats exist, however. In exon 3, for example, it is possible that the transient mispairing of the two imperfect repeat units at some stage during DNA replication gives rise to the looping out and excision of the non-homologous bases. Alternatively, the first repeat is a quasi repeat which might have been copied producing the deletion. Another mechanism involves short runs of the same bases. In exon 17, for example, one of the two 'AA' nucleotides is deleted. At this locus, transient misalignment of the newly synthesised strand by one base pair could have resulted in the excision of the missed, unpaired 'A'. Twenty-seven out of 60 deletions studied by Krawczak and Cooper (1991) contained inverted repeats which either flanked or span the deletions. Frequent co-occurrence, as in the case of exon 17 deletion reported here, of inverted repeats and direct repeats at sites of deletions were also noted (Krawczak & Cooper, 1991). That these sequences can form stem and loop structures tends to suggest that they are potential hot spots for structural rearrangements, possibly also generating structures recognised by eukaryotic endonucleases such as topoisomerase I (Nalbantoglu *et al.*, 1986). A mechanism involving the formation of a hairpin loop was suggested by Shew *et al.* (1990) in a deletion they reported in the RB1 gene.

The structural motifs we observed around the two deletions were also present around the insertion in exon 13 suggesting that insertions may arise by a similar mechanism. Not only is the insertion within 8–9 bp direct repeats, parts of which are symmetrical, but this particular repeat sequence is also present in exon 10, 11 and 27 of the RB1 gene (T'Ang *et al.*, 1989). The simplest mechanism for this insertion would involve misalignment of the newly synthesised DNA over the repeats and then re-alignment or the symmetrical element flanking the insertion (AGGATAATAGGA) giving rise to secondary structures such as Moebius loops (Cooper & Krawczak, 1991) which facilitate an insertion. We have previously reported another insertion in exon 20 of RB1 which is associated with a 4 bp direct repeat (Hogg *et al.*, 1992). In the analysis of Cooper and Krawczak (1991) insertions from various genetic loci were also found to be associated with direct repeats and/or symmetrical elements which suggests the involvement of these structures in insertion mutagenesis.

Deletions and insertions also appear to occur near to the sequence TG A/G A/G G/T A/C (Cooper & Krawczak, 1991; Krawczak & Cooper, 1991) which is homologous to the putative arrest sites for DNA polymerase α. Similar sequences were present in all three mutations reported here with the initial TG A being invariant. The sequence observed near exon 13 insertion contained one mismatch in the other three bases whereas those observed in exon 3 and 17 contained two mismatches (Figure 5). Similar sequences exist in other deletions and insertions in RB1 (Dunn *et al.*, 1989; Hogg *et al.*, 1992; Lohmann *et al.*, 1992; Yandell *et al.*, 1989). It has been suggested that arrest of DNA synthesis at such sites might, by providing a stable single stranded substrate, lead to deletions and insertions by a variety of mechanisms (Cooper & Krawczak, 1991; Krawczak & Cooper, 1991).

We have, thus, identified similar sequence motifs around different mutations in the RB1 gene. It will be interesting to see whether a consistent pattern will emerge when more mutations are characterised in this gene which might lead to the identification of areas more likely to give rise to mutations.

There have been several reports (Horowitz *et al.*, 1990; Mori *et al.*, 1990; Murakami *et al.*, 1991; Weir-Thompson *et al.*, 1991) where mutations in RB1 apparently result in missense or termination codon mutations. Many of these were from non-Rb tumours and identified because it was possible to screen mRNA from these tumours. Although the 2 bp deletion in exon 3 in RBF64 is 9 bp upstream of the splice donor site for exon 3 it may influence the splicing efficiency at this site or alternatively, exon 3 might be spliced out all together from the mature mRNA. The latter possibility, however, affects the reading frame and results in a stop codon further down in exon 4. There is evidence from the study of other genes that a change in the local sequence environment can affect splicing patterns (Reed & Maniatis, 1986; Steingrimsdottir *et al.*, 1992). Because of efficient ophthalmological screening of children born to Rb patients, their tumours are treated *in situ* and so are rarely available to study the mRNA. Whether these mutations adjacent to splice sites affect mRNA processing will require *in vitro* studies.

DNA from the three families reported here were part of a series from Rb patients and Rb tumours some of which had distinctive phenotypes (Onadim *et al.*, 1992*b*; Hogg *et al.*, 1993). SSCP analysis proved to be an efficient way of screening for mutations in the RB1 gene. With the clinical application of mutation screening, mutant gene carriers can be detected in the absence of a family history. For the families whose causative mutations have been identified, on the other hand, unequivocal screening of unaffected individuals in the family and the fetuses of affected individuals makes genetic counselling straightforward.

We would like to thank Sheila Giles for assistance in typing the manuscript and preparing the graphics and Dr I. Goldsmith and his group at the ICRF for preparing the oligonucleotides. Z.O. and A.H. were supported by a grant from the David Allen Retinoblastoma Appeal.

**References**

BLANQUET, V., CREAU-GOLDBERG, N., DE GROUCHY, J. & TURLEAU, C. (1991). Molecular detection of constitutional deletions in patients with retinoblastoma. *Am. J. Med. Genet.*, **39**, 355-361.

CANNING, S. & DRYJA, T.P. (1989). Short direct repeats at the breakpoints of deletions of the retinoblastoma gene. *Proc. Natl Acad. Sci. USA*, **86**, 5044-5048.

CAVENEE, W., DRYJA, T.P., PHILLIPS, R.A., BENEDICT, W.F., GODBOUT, R., GALLIE, B.L., MURPHREE, A.L., STRONG, L.C. & WHITE, R. (1983). Expression of recessive alleles by chromosomal mechanisms in retinoblastoma. *Nature*, **305**, 779-784.

CAVENEE, W.K., HANSEN, M.F., NORDENSKJOLD, M., KOCK, E., MAUMENEE, I., SQUIRE, J.A., PHILLIPS, R.A. & GALLIE, B.L. (1985). Genetic origin of mutations predisposing to retinoblastoma. *Science*, **228**, 501-503.

COOPER, D.N. & KRAWCZAK, M. (1991). Mechanisms of insertional mutagenesis in human genes causing genetic disease. *Hum. Genet.*, **87**, 409-415.

COWELL, J.K. (1992). Tumour suppressor genes. *Annal. Oncol.*, **3**, 693-698.

COWELL, J.K. & HOGG, A. (1992). The genetics and cytogenetics of retinoblastoma. *Cancer Genet. Cytogenet.*, **64**, 1-11.

COWELL, J.K., JAY, M., RUTLAND, P. & HUNGERFORD, J. (1987). An assessment of the usefulness of electrophoretic variants of esterase-D in antenatal diagnosis of retinoblastoma in the United Kingdom. *Br. J. Cancer*, **55**, 661-664.

DEAN, M., WHITE, M.B., AMOS, J., GERRARD, B., STEWART, C., KHAW, K.-T. & LEPPERT, M. (1990). Multiple mutations in highly conserved residues are found in mildly affected cystic fibrosis patients. *Cell*, **61**, 863-870.

DRAPER, G.J., SANDERS, B.M., BROWNBILL, P.A. & HAWKINS, M.M. (1992). Patterns of risk of hereditary retinoblastoma and applications to genetic counselling. *Br. J. Cancer*, **66**, 211-219.

DUNN, J.M., PHILLIPS, R.A., BECKER, A. & GALLIE, B.L. (1988). Identification of germline and somatic mutations affecting the retinoblastoma gene. *Science*, **241**, 1797-1800.

DUNN, J.M., PHILLIPS, R.A., ZHU, X., BECKER, A. & GALLIE, B.L. (1989). Mutations in the RB1 gene and their effects on transcription. *Mol. Cell Biol.*, **9**, 4596-4604.

EFSTRATIADIS, A., POSAKONY, J.W., MANIATIS, T., LAWN, R.M., O'CONNELL, C., SPRITZ, R.A., DERIEL, J.K., FORGET, B.G., WEISSMAN, S.M., SLIGHTOM, J.L., BLECHL, A.E., SMITHIES, O., BARALLE, F.E., SHOULDERS, C.C. & PROUDFOOT, N.J. (1980). The structure and evolution of the human $\beta$ globin gene family. *Cell*, **21**, 653-668.

FARABAUGH, P.J., SCHMEISSNER, U., HOFER, M. & MILLER, J.H. (1978). Genetic studies of the *lac* repressor VII. On the molecular nature of spontaneous hotspots in the *lac* I gene of *Escherichia coli*. *J. Mol. Biol.*, **126**, 847-863.

FRIEND, S.H., BERNARDS, R., ROGELI, S., WEINBERG, R.A., RAPAPORT, J.M., ALBERT, D.M. & DRYJA, T.P. (1986). A human DNA segment with properties of the gene that predisposes to retinoblastoma and osteosarcoma. *Nature*, **323**, 643-646.

FUNG, Y.T., MURPHREE, A.L., T'ANG, A., QUIAN, J., HINRICHS, S.H. & BENEDICT, W.F. (1987). Structural evidence for the authenticity of the human retinoblastoma gene. *Science*, **236**, 1657-1661.

GODDARD, A.D., BALAKIER, H., CANTON, M., DUNN, J., SQUIRE, J., REYES, E., BECKER, A., PHILLIPS, R.A. & GALLIE, B.L. (1988). Infrequent genomic rearrangement and normal expression of the putative RB1 gene in retinoblastoma tumours. *Mol. Cell Biol.*, **8**, 2082-2088.

GODDARD, A.D., PHILLIPS, R.A., GREGER, V., PASSARGE, E., HOPPING, W., ZHU, X., GALLIE, B.L. & HORSTHEMKE, B. (1990). Use of the RB1 cDNA as a diagnostic probe in retinoblastoma families. *Clin. Genet.*, **37**, 117-126.

HASHIMOTO, T., TAKAHASHI, R., YANDELL, D.W., XU, H.-J., XU, S.-X., GUNNELL, S. & BENEDICT, W.F. (1991). Characterisation of intragenic deletions in two sporadic germinal mutation cases of retinoblastoma resulting in abnormal gene expression. *Oncogene*, **6**, 463-469.

HOGG, A., ONADIM, Z., BAIRD, P.N. & COWELL, J.K. (1992). Detection of heterozygous mutations in the RB1 gene in retinoblastoma patients using single strand conformation polymorphism (SSCP) analysis and polymerase chain reaction sequencing. *Oncogene*, **7**, 1444-1451.

HOGG, A., BIA, B., ONADIM, Z. & COWELL, J.K. (1993). Molecular mechanisms of oncogenic mutations in tumours from patients with bilateral and unilateral retinoblastoma. *Proc. Natl Acad. Sci. USA*, (in press).

HOROWITZ, J.M., PARK, S.-H., BOGENMANN, E., CHENG, J.-C., YANDELL, D.W., KAYE, F.J., MINNA, J.D., DRYJA, T.P. & WEINBERG, R.A. (1990). Frequent inactivation of the retinoblastoma anti-oncogene is restricted to subset of human tumour cells. *Proc. Natl Acad. Sci. USA*, **87**, 2775-2779.

KLOSS, K., WAHRISCH, P., GREGER, V., MESSMER, E., FRITZE, H., HOPPING, W., PASSARGE, E. & HORTSHEMKE, B. (1991). Characterisation of deletions at the retinoblastoma locus in patients with bilateral retinoblastoma. *Am. J. Med. Genet.*, **39**, 196-200.

KNUDSON, A.G. (1971). Mutation and cancer: statistical study of retinoblastoma. *Proc. Natl Acad. Sci. USA*, **68**, 820-823.

KRAWCZAK, M. & COOPER, D.N. (1991). Gene deletions causing human genetic disease: mechanisms of mutagenesis and the role of the local DNA sequence environment. *Hum. Genet.*, **86**, 425-441.

LOHMANN, D., HORSTHEMKE, B., GILLESSEN-KAESBACH, G., STEFANI, F.H. & HOFLER, H. (1992). Detection of small RB1 gene deletions in retinoblastoma by multiplex PCR and high-resolution gel electrophoresis. *Hum. Genet.*, **89**, 49-53.

MAZARS, R., PUJOL, P., MAUDELONDE, T., JEANTEUR, P. & THEILLET, T. (1991). p53 mutations in ovarian cancer: a late event? *Oncogene*, **6**, 1685-1690.

MCGEE, T.L., YANDELL, D.W. & DRYJA, T.P. (1989). Structure and partial genomic sequence of the human retinoblastoma susceptibility gene. *Gene*, **80**, 119-128.

MITCHELL, C.D. & COWELL, J.K. (1989). Predisposition to retinoblastoma due to a translocation within the 4.7R locus. *Oncogene*, **4**, 253-257.

MORI, N., YOKOTA, J., AKIYAMA, T., SAMESHIMA, Y., OKAMOTO, A., MIZOGUCHI, H., TOYOSHIMA, K., SUGIMURA, T. & TERADA, M. (1990). Variable mutations of the RB gene in small-cell lung carcinoma. *Oncogene*, **5**, 1713-1717.

MURAKAMI, Y., KATAHIRA, M., MAKINO, R., HAYASHI, K., HIROHASHI, S. & SEKIYA, T. (1991). Inactivation of the retinoblastoma gene in a human lung carcinoma cell line detected by single-strand conformation polymorphism analysis of the polymerase chain reaction product of cDNA. *Oncogene*, **6**, 37-42.

NALBANTOGLU, J., HARTLEY, D., PHEAR, G., TEAR, G. & MEUTH, M. (1986). Spontaneous deletion formation at the *aprt* locus of hamster cells: the presence of short sequence homologies and dyad symmetries at deletion termini. *EMBO*, **5**, 1199-1204.

ONADIM, Z. & COWELL, J.K. (1991). Application of PCR amplification from paraffin embedded tissue sections to linkage analysis in familial retinoblastoma. *J. Med. Genet.*, **28**, 312-316.

ONADIM, Z., HOGG, A., BAIRD, P.N. & COWELL, J.K. (1992*b*). Oncogenic point mutations in exon 20 of the RB1 gene in families showing incomplete penetrance and mild expression of the retinoblastoma phenotype. *Proc. Natl Acad. Sci. USA*, **89**, 6177-6181.

ONADIM, Z., HUNGERFORD, J. & COWELL, J.K. (1992*a*). Follow-up of retinoblastoma patients having prenatal and perinatal predictions for mutant gene carrier status using intragenic polymorphic probes from the RB1 gene. *Br. J. Cancer*, **65**, 711–716.

ONADIM, Z., MITCHELL, C.D., RUTLAND, P.C., BUCKLE, B.G., JAY, M., HUNGERFORD, J.L., HARPER, K. & COWELL, J.K. (1990). Application of intragenic DNA probes in prenatal screening for retinoblastoma gene carriers in the United Kingdom. *Arch. Dis. Child.*, **65**, 651–656.

ORITA, M., SUZUKI, Y., SEKIYA, T. & HAYASHI, K. (1989). Rapid and sensitive detection of point mutations and DNA polymorphisms using the polymerase chain reaction. *Genomics*, **5**, 874–879.

REED, R. & MANIATIS, T. (1986). A role for exon sequences and splice-site proximity in splice-site selection. *Cell*, **46**, 681–690.

ROTH, D.B., PORTER, T.N. & WILSON, J.H. (1985). Mechanisms of non-homologous recombination in mammalian cells. *Mol. Cell Biol.*, **5**, 2599–2607.

SCHEFFER, H., MEERMAN, G.J., KRUISE, Y.C.M., VAN DEN BERG, A.H.M., PENNING, D.P., TAN, K.E.W.P., DER KINDEREN, D.J. & BUYS, C.H.C.M. (1989). Linkage analysis of families with hereditary retinoblastoma; non-penetrance of mutation, revealed by combined use of markers within and flanking the RB1 gene. *Am. J. Hum. Genet.*, **45**, 252–260.

SHEW, J.-Y., CHEN, P.-L., BOOKSTEIN, R., LEE, E.Y.-H.P. & LEE, W.-H. (1990). Deletion of splice donor site ablates expression of the following exon and produces an unphosphorylated Rb protein unable to bind SV40 T antigen. *Cell Growth Different.*, **1**, 17–25.

STEINGRIMSDOTTIR, H., ROWLEY, G., DORADO, G., COLE, J. & LEHMANN, A.R. (1992). Mutations which alter splicing in the human hypoxanthine-guanine phosphoribosyl transferase gene. *Nucleic Acid Res.*, **20**, 1201–1208.

T'ANG, A., WU, K.-J., HASHIMOTO, T., LIU, W.-Y., TAKAHASHI, R., SHI, X.-H., MIHARA, K., ZHANG, F.-H., CHEN, Y.-Y., DU, C., QIAN, J., LIN, Y.-G., MURPHREE, A.L., QIU, W.-R., THOMPSON, T.F.B.W. & FUNG, Y.-K. (1989). Genomic organization of the human retinoblastoma gene. *Oncogene*, **4**, 401–407.

VOGEL, W. (1979). The genetics of retinoblastoma. *Hum. Genet.*, **52**, 1–54.

WEAVER, D.T. & DEPAMPHILIS, M.L. (1982). Specific sequences in native DNA that arrest synthesis by DNA polymerase α. *J. Biol. Chem.*, **257**, 2075–2086.

WEIR-THOMPSON, E., CONDIE, A., LEONARD, R.C.F. & PROSSER, J. (1991). A familial RB1 mutation detected by the HOT technique is homozygous in a second primary neoplasm. *Oncogene*, **6**, 2353–2356.

WIGGS, J., NORDENSKJELD, M., YANDELL, D., RAPAPORT, J., GRONDIN, V., JANSON, M., WERELIUS, B., PETERSEN, R., CRAFT, A., RIEDEL, K., LIEBERFARB, R., WALTON, D., WILTON, W. & DRYJA, T.P. (1988). Prediction of the risk of hereditary retinoblastoma using DNA polymorphisms within the retinoblastoma gene. *New Eng. J. Med.*, **318**, 151–157.

YANDELL, D.W., CAMPBELL, T.A., DAYTON, S.H., PETERSEN, R., WALTON, D., LITTLE, J.B., MCCONKIE-ROSELL, A., BUCKLEY, E.G. & DRYJA, T.P. (1989). Oncogenic point mutations in the human retinoblastoma gene: their application to genetic counselling. *New Eng. J. Med.*, **321**, 1689–1695.

YANDELL, D.W. & DRYJA, T.P. (1989). Detection of DNA sequence polymorphisms by enzymatic amplification and direct genomic sequencing. *Am. J. Hum. Genet.*, **45**, 547–555.

ZHU, X., DUNN, J.M., GODDARD, A.D., SQUIRE, J.A., BECKER, A., PHILLIPS, R.A. & GALLE, B.L. (1992). Mechanisms of loss of heterozygosity in retinoblastoma. *Cytogenet. Cell Genet.*, **59**, 248–252.